# Supplementary material for: MetaRibo-Seq measures translation in microbiomes
Source: Nat Commun. 2020 Jun 29;11:3268. doi: 10.1038/s41467-020-17081-z (PMC7324362; doi:10.1038/s41467-020-17081-z)
Supplement: Supplementary file 10 — Supplementary Data 7 [file 41467_2020_17081_MOESM10_ESM.zip › File2/Confidence_High_Taxonomy/111223_out.krona.html]

Javascript must be enabled to view this page.

members
magnitude
magnitudeUnassigned
count
unassigned
taxon
rank

111223\_out

3

3
superkingdom
2

1239
phylum
3

3
class
186801

186802
3
order

3
family
186803

841
1
3
genus

SRS014923\_contig\_number\_contig-100\_5292.44770

1262943

SRS075716\_contig\_number\_contig-100\_3100.3101
species
1

1897018
1
species

SRS148721\_contig\_number\_23619
